# The importance of PrEP persistence in preventing HIV infections on PrEP

**DOI:** 10.1002/jia2.25578

**Published:** 2020-08-26

**Authors:** Matthew A Spinelli, Nicole Laborde, Patrick Kinley, Ryan Whitacre, Hyman M Scott, Nicole Walker, Albert Y Liu, Monica Gandhi, Susan P Buchbinder

**Affiliations:** ^1^ Division of HIV, ID, and Global Medicine University of California San Francisco CA USA; ^2^ San Francisco Department of Public Health San Francisco CA USA; ^3^ Department of Anthropology and Sociology The Graduate Institute Geneva Geneva Switzerland

**Keywords:** PrEP, HIV seroconversion, adherence, PrEP persistence, mixed methods, PrEP discontinuation

1

We are pleased to learn that our paper’s themes resonated with other contexts such as the authors’ pre‐exposure prophylaxis (PrEP) programme in Sydney, Australia. We are also interested to learn that there is similarly limited awareness and resources supporting PrEP 2‐1‐1/on‐demand dosing [1] in Australia. Anecdotally, several of our PrEP patients are considering transitioning to PrEP 2‐1‐1 dosing during ongoing shelter‐in‐place in response to the COVID‐19 epidemic, but are worried their clinicians may not support it.

We agree that stigma related to PrEP or sexual behaviour [2, 3, 4] is an important factor that may interfere with PrEP persistence (consistent use of PrEP during periods with potential HIV exposure) [5]. Participants in this analysis did not report stigma related to being a man who has sex with men (MSM) as a factor in their PrEP gaps, but two participants discussed stigma related to the image of PrEP as a medication for people with multiple sexual partners, and at least one participant feared disclosure of her PrEP use to her partner as she felt it might imply she had outside partners [6].

We agree with the authors that PrEP uptake is an important component of the PrEP cascade, and will be crucial towards meeting global targets to reduce HIV transmission [7, 8]. As the design of this analysis involved a sample of all individuals who had used PrEP at any point in our health system, we were not able to interview individuals who HIV seroconverted and had never used PrEP before. In an analysis within another integrated health system in Northern California, barriers to PrEP use among those who were aware of PrEP prior to HIV diagnosis but never started it included: cost/insurance issues, perceived low risk for HIV acquisition and concerns about being stigmatized if they used PrEP [9]. In San Francisco in 2017, approximately 40% of MSM had used PrEP in the last six months, whereas 50% reported condomless anal sex over the same time period [10]. We, however, found in a prior analysis that the relative risk of PrEP discontinuation is unfortunately increasing over time, potentially because later versus early adopters are more ambivalent about using and remaining on PrEP [11]. As more individuals try PrEP in our jurisdiction and others, we will need to turn our focus towards intervenable factors that can support PrEP persistence. In order to maximize PrEP’s prevention efficacy, individuals will need to remain on PrEP with adequate adherence during periods of HIV exposure. We agree that there is no time to waste in addressing potential barriers to implementation of this highly effective, but still underutilized, HIV prevention intervention.

### COMPETING INTEREST

The authors have no conflicts of interest to report.

### AUTHORS’ CONTRIBUTIONS

MAS drafted the letter. NL, PK, RW, HMS, NW, AYL, MG and SPB revised the letter for content. All authors have read and approved the final manuscript of the letter.

REFERENCES1

Molina
JM
, 
Charreau
I
, 
Spire
B
, 
Cotte
L
, 
Julie Chas
J
, 
Catherine Capitant
C
, et al. Efficacy, safety, and effect on sexual behaviour of on‐demand pre‐exposure prophylaxis for HIV in men who have sex with men: an observational cohort study. Lancet HIV. 2017;4(9):e402–e10.10.1016/S2352-3018(17)30089-9287472742

Hess
KM
, 
Crawford
J
, 
Eanes
A
, 
Felner
JK
, 
Mittal
ML
, 
Smith
LR
,, et al. Reasons why young men who have sex with men report not using HIV Pre‐exposure prophylaxis: perceptions of burden, need, and safety. AIDS Patient Care STDS. 2019;33(10):449–54.3158485610.1089/apc.2019.0150PMC67851683

Siegler
AJ
, 
Wiatrek
S
, 
Mouhanna
F
,
Amico
KR
, 
Dominguez
K
, 
Jones
J
, et al. Validation of the HIV pre‐exposure prophylaxis stigma scale: performance of Likert and semantic differential scale versions. AIDS Behav. 2020
10.1007/s10461-020-02820-6
PMC7423865321574904

Brooks
RA
, 
Cabral
A
, 
Nieto
O
, 
Fehrenbacher
A
, 
Landrian
A
. Experiences of pre‐exposure prophylaxis stigma, social support, and information dissemination among black and Latina transgender women who are using pre‐exposure prophylaxis. Transgend Health. 2019;4(1):188–96.3148213410.1089/trgh.2019.0014PMC67161885

Spinelli
MA
, 
Buchbinder
SP
. PrEP Persistence is a critical issue in PrEP implementation. Clin Infect Dis. 2019:ciz896
10.1093/cid/ciz896
PMC7384308315096036

Spinelli
MA
, 
Laborde
N
, 
Kinley
P
, 
Whitacre
R
, 
Scott
H
, 
Walker
N
, et al. Missed opportunities to prevent HIV infections among pre‐exposure prophylaxis users: a population‐based mixed methods study, San Francisco, United States. J Int AIDS Soc. 2020;23:e25472.3229433810.1002/jia2.25472PMC71592497

Baggaley
R
, 
Dalal
S
, 
Johnson
C
, 
Macdonald
V
, 
Mameletzis
I
, 
Rodolph
M
, et al. Beyond the 90–90‐90: refocusing HIV prevention as part of the global HIV response. J Int AIDS Soc. 2016;19:21348.2798927110.7448/IAS.19.1.21348PMC51650838

Krishnaratne
S
, 
Hensen
B
, 
Cordes
J
, 
Enstone
J
, 
Hargreaves
JR
. Interventions to strengthen the HIV prevention cascade: a systematic review of reviews. Lancet HIV. 2016;3(7):e307–17.10.1016/S2352-3018(16)30038-8273652059

Marcus
JL
, 
Hurley
LB
, 
Dentoni‐Lasofsky
D
, 
Ellis
CG
, 
Silverberg
MJ
, 
Slome
S
, et al. Barriers to preexposure prophylaxis use among individuals with recently acquired HIV infection in Northern California. AIDS Care. 2019;31(5):536–44.3030494210.1080/09540121.2018.1533238PMC640823510
San Francisco Department of Public Health
. San Francisco HIV Epidemiology Annual Report 2018. San Francisco Department of Public Health HIV Epidemiology Section. 2019.11

Spinelli
MA
, 
Scott
HM
, 
Vittinghoff
E
, 
Liu
AY
, 
Gonzalez
R
, 
Morehead‐Gee
A
, et al. Missed visits are associated with future pre‐exposure prophylaxis (PrEP) discontinuation among PrEP users in a municipal primary care health network. Open Forum Infect Dis. 2019;6:ofz101.3094954010.1093/ofid/ofz101PMC6441570
